# Validation of Preoperative Neoadjuvant Bevacizumab Therapy for Newly Diagnosed Glioblastoma via Comparative Analyses with Propensity Score Matching

**DOI:** 10.3390/cancers18030488

**Published:** 2026-02-01

**Authors:** Yohei Yamamoto, Akihiko Teshigawara, Ryota Tamura, Jun Takei, Yukina Morimoto, Kyoichi Tomoto, Yasuharu Akasaki, Yuzuru Hasegawa, Yuichi Murayama, Keisuke Miyake, Hikaru Sasaki, Toshihide Tanaka

**Affiliations:** 1 Department of Neurosurgery, The Jikei University School of Medicine, Kashiwa Hospital, Chiba 277-8657, Japan; yohei_1109@yahoo.co.jp (Y.Y.); de.apipiko817tepigawara@gmail.com (A.T.); annindof72@gmail.com (K.T.); yuz-yuz@next.odn.ne.jp (Y.H.); 2 Department of Neurosurgery, The Jikei University School of Medicine, West Medical Center, Tokyo 201-8601, Japan; 3 Department of Neurosurgery, Keio University School of Medicine, Tokyo 160-8582, Japan; moltobello-r-610@keio.jp (R.T.); yukinaxnashiko@yahoo.co.jp (Y.M.); hssasaki@tdc.ac.jp (H.S.); 4 Department of Neurosurgery, The Jikei University School of Medicine, Tokyo 105-8471, Japan; jun.takei1986@gmail.com (J.T.); akasaki@jikei.ac.jp (Y.A.); ymurayama@jikei.ac.jp (Y.M.); 5 Department of Neurosurgery, Kagawa University Graduate School of Medicine, Kagawa 761-0793, Japan; miyake.keisuke@kagawa-u.ac.jp; 6 Department of Neurosurgery, Tokyo Dental College Ichikawa General Hospital, Chiba 272-8513, Japan

**Keywords:** angiogenesis, expanding perifocal edema, glioblastoma, neoadjuvant bevacizumab, overall survival

## Abstract

Background/Objectives: The role of preoperative neoadjuvant bevacizumab (neoBev) in newly diagnosed glioblastoma (GB) remains controversial. We evaluated its impact on perioperative and survival outcomes and explored imaging-based associations with prognosis. Methods: We retrospectively analyzed 33 patients with newly diagnosed GB who received preoperative neoBev (10 mg/kg; surgery performed 21–30 days later). A matched control cohort of 33 patients treated with standard chemoradiotherapy was selected using propensity score matching. Changes in Karnofsky Performance Status (KPS), extent of resection, and volumetric alterations on gadolinium-enhanced T1-weighted MRI (T1Gd) and FLAIR were assessed. Progression-free survival (PFS) and overall survival (OS) were compared. Results: All patients had GB (31 IDH-wild-type, 2 IDH-mutant). Median KPS improved after neoBev (71 to 83), while no improvement was observed in controls. After matching, median PFS and OS did not differ between the neoBev and control groups (7.4 vs. 8.6 months and 13.4 vs. 13.8 months, respectively). Exploratory analyses in the neoBev cohort showed that greater T1Gd volume reduction was associated with improved PFS and OS, whereas FLAIR changes were not. Conclusions: Preoperative neoBev did not improve survival compared with standard chemoradiotherapy. However, it was associated with improved functional status, and exploratory imaging findings suggested associations between early radiographic response and outcome. NeoBev should not be used routinely but may have a contextual role in selected patients with perioperative challenges.

## Simple Summary

Bevacizumab is widely used in the treatment of glioblastoma, although its role in newly diagnosed disease remains controversial. In this study, we evaluated the clinical impact of preoperative neoadjuvant bevacizumab by comparing perioperative and survival outcomes with those of patients treated with standard chemoradiotherapy using propensity score matching. Preoperative bevacizumab did not improve progression-free or overall survival compared with standard therapy. However, exploratory imaging analyses suggested that a greater reduction in contrast-enhanced tumor volume on magnetic resonance imaging was associated with more favorable survival outcomes, although these findings require external validation. Significantly, preoperative bevacizumab was associated with improved postoperative functional status, suggesting a potential role in optimizing perioperative conditions. These results indicate that neoadjuvant bevacizumab should not be used routinely in newly diagnosed glioblastoma, but may have a contextual role in selected patients for whom perioperative factors—such as extensive edema, lower performance status, or tumor involvement of eloquent brain regions—complicate immediate surgical management.

## 1. Introduction

Vascular endothelial growth factor (VEGF) promotes endothelial proliferation and increases vascular permeability. As a monoclonal antibody against VEGF, bevacizumab (Bev) can reduce both tumor volume and perifocal edema in glioblastoma (GB). This may lead to reduced contrast enhancement and decreased hyperintensity volume on fluid-attenuated inversion recovery (FLAIR) imaging [1]. These effects are consistent with prior clinical imaging studies in glioblastoma, which showed that pharmacologic VEGF receptor blockade rapidly normalized tumor vasculature and alleviated edema [2]. These effects might lessen the surgical burden by decreasing vascularity and improving cerebral edema. Indeed, neoBev therapy has been shown to reduce tumor size and enhance therapeutic response in several cancers [3,4,5,6].

Based on a prior prospective Phase II study, we observed significant reductions in tumor vascularity and brain swelling without severe adverse events [1]. Given the volume reduction rates achieved with preoperative neoBev, a larger decrease in volume seen on T1-weighted imaging with gadolinium enhancement (T1Gd) compared to FLAIR was associated with a tendency toward longer median overall survival (mOS) after neoBev. These results suggest that the volume reduction in FLAIR abnormalities may be a pseudo-response. That volume reduction on T1Gd offers a more reliable indicator of neoBev response, which could be linked to mOS [1]. The effect of neoBev on FLAIR image changes has not been definitively identified as a reliable marker.

Development of surgical techniques with a supporting system and therapeutic stratification based on molecular diagnosis has been shown to improve clinical outcomes for GB [7,8]. Preoperative neoBev could help decrease the difficulty and morbidity of tumor resection, particularly in specific situations, including bulky hypervascular tumors with a complex network of feeding arteries with edema extending into eloquent areas. However, the initial response to one-shot Bev did not always reflect improvement in OS.

The initial exploratory evaluation of neoBev in resectable GB was conducted as a single-arm study, primarily to establish the feasibility, safety, and short-term radiographic effects before surgery. In contrast, the present investigation was designed as a retrospective comparative analysis to determine whether the clinical applicability of neoBev could be extended beyond feasibility alone. To contextualize the potential benefits, we compared neoBev with conventional standard therapy (comprising radiation therapy [RT] with concomitant temozolomide [TMZ]) using propensity score matching (PSM) to approximate a balanced comparison in selected clinical scenarios.

In addition, the additive benefit of neoBev was considered to alter the tumor microenvironment, including oxygenation and stemness, before and after Bev therapy in newly diagnosed GB using paired samples [9,10,11]. As reported previously, immune regulatory cells and checkpoint molecules were also compared during Bev therapy [10,12]. In neoBev (i.e., during the effectiveness of Bev), tumor oxygenation, a decrease in stem cell population, and an immunosupportive tumor microenvironment (TME) were observed, compared with naïve and refractory states to Bev [9,10,12]. We hypothesized that altering the TME with Bev could enhance the efficacy of postoperative adjuvant RT and TMZ therapy, thereby prolonging progression-free survival (PFS) and OS.

This study aimed to evaluate the clinical outcomes of neoBev compared to the standard protocol, which includes surgery and postoperative concurrent RT and TMZ, in patients with newly diagnosed GB. However, whether neoBev offers clinical benefits beyond facilitating surgery (particularly in comparison with modern chemoradiation) remains unknown. No previous research has directly assessed the effects of NeoBev on survival in patients with newly diagnosed resectable GB.

## 2. Materials and Methods

### 2.1. Patient Registration

This was a multi-institutional retrospective cohort study. Patient eligibility, treatment indications, and perioperative management followed the protocol described in our previous report (Tanaka et al., 2024) [1], from which the present secondary analysis was derived. We included 33 consecutive patients who received neoBev at The Jikei University School of Medicine, Keio University Hospital, and Kagawa University Hospital between January 2015 and August 2024. Eligibility criteria were: (i) newly diagnosed GB; (ii) intention to proceed with maximal safe resection after neoBev; and (iii) availability of pre- and post-neoBev MRI suitable for volumetric assessment T1-Gd and FLAIR). Patients with prior cranial RT or TMZ, insufficient imaging, or inadequate clinical follow-up were excluded. When molecular data were available, diagnoses were harmonized with the 2021 World Health Organization classification [13]. Cases without molecular testing were classified according to the integrated histopathological criteria in effect at the time of treatment.

As a control cohort, we used institutional databases to identify 136 consecutive patients with newly diagnosed GB who underwent standard therapy without neoBev at The Jikei University School of Medicine during the same period. Baseline clinical and radiological variables were abstracted uniformly across cohorts. PSM was prespecified to mitigate baseline imbalances and is detailed in the Section 2.4.

A total of 169 patients were screened, of whom 169 met the inclusion criteria. No patients were excluded for missing survival data, and all included patients were followed until death or the last follow-up. Minimum follow-up at the time of analysis was 15 months. Survival endpoints and censoring rules are described below. In clinical practice, selection for neoadjuvant bevacizumab was influenced not only by predefined eligibility criteria but also by pragmatic considerations related to surgical risk. NeoBev was preferentially considered in patients with marked mass effect and extensive peritumoral edema on MRI, prominent discrepancy between contrast-enhancing tumor volume and FLAIR hyperintensity, tumors involving or adjacent to eloquent regions, and/or significant neurological deficits such as hemiparesis or aphasia.

All patients (or their legal representatives) provided written informed consent for treatment and the use of de-identified data. The study protocol was approved by the institutional review boards of all participating centers and conducted in accordance with the principles of the Declaration of Helsinki (2013 revision) [14].

### 2.2. Treatment

Preoperative neoBev was administered intravenously at a dose of 10 mg/kg on day 0, as previously described [1]. Maximal safe resection was then scheduled between days 21 and 30 after neoBev, provided no clinical or wound-healing concerns were present. After resection, patients received RT with concurrent and adjuvant TMZ according to the standard published protocol [15] (i.e., a Stupp-type regimen), as per institutional practice.

Extent of resection (EOR) was assessed using T1Gd performed within 72 h after surgery. EOR was classified as: gross total resection (GTR), no measurable residual enhancement; subtotal resection (STR), defined as EOR ≥ 90%, or partial resection (PR), defined as EOR < 90%. Perioperative management (including steroid tapering and venous thromboembolism prophylaxis) followed institutional standards, with particular focus on wound healing and hemorrhagic complications due to prior anti-VEGF exposure.

The timing of surgery after neoadjuvant bevacizumab was determined based on pharmacokinetic and biological considerations. Bevacizumab has a reported serum half-life of approximately 20 days, and vascular endothelial growth factor (VEGF) and VEGF receptor signaling are known to play critical roles in wound healing, with bimodal peaks observed approximately 2 and 4 weeks after surgery. To balance the persistence of anti-edema and vascular normalization effects while minimizing potential interference with wound healing, surgery was scheduled 21–30 days after bevacizumab administration. This interval was selected to allow partial drug washout while preserving its perioperative benefits, consistent with prior clinical experience and published reports [1].

### 2.3. Assessments

PFS and OS were assessed in both the NeoBev and Control groups according to Response Assessment in Neuro-Oncology (RANO) criteria. Both median PFS (mPFS) and mOS were calculated from the date of diagnosis (or surgery) to radiographic or clinical progression, or death, whichever occurred first.

Karnofsky Performance Status (KPS) was recorded preoperatively, after neoBev administration, and postoperatively (early recovery or discharge). Changes in KPS (ΔKPS) were analyzed within and between groups to evaluate functional improvement. Preoperative KPS was assessed at diagnosis prior to any neoadjuvant treatment, and postoperative KPS was evaluated after surgical recovery in both groups; KPS was not assessed during the neoadjuvant interval.

Baseline variables—including age, sex, preoperative KPS, tumor location (eloquent vs. non-eloquent), and EOR—were compared between the NeoBev and Control groups. Intergroup imbalances were adjusted using PSM as described in the Section 2.4, and associations with mPFS and mOS were then examined.

Serial brain MRI examinations were obtained at prespecified time points (baseline, post-neoBev, and 72 h postoperatively), as previously described [1]. MRI sequences included T1Gd and FLAIR. In the NeoBev group, volumetric changes in the contrast-enhancing tumor and peritumoral edema were quantified using semi-automated segmentation.

Cutoff values for volumetric change were evaluated using receiver operating characteristic (ROC) analysis to generate hypotheses. Because of the stepwise nature of ROC curves in this limited sample, optimal thresholds were defined by the shortest Euclidean distance to the upper-left corner of the ROC space (closest-to-(0,1) method), prioritizing a balanced trade-off between sensitivity and specificity. Patients were then classified as good or poor responders according to these thresholds, and mPFS/mOS were compared between responder subgroups. In sensitivity analyses, cutoffs based on the Youden index yielded directionally consistent results. Blinding was not feasible due to the retrospective nature of the study. Because these ROC-derived thresholds were identified post hoc in a limited sample, they should be interpreted cautiously and considered hypothesis-generating.

### 2.4. Statistical Analyses

Continuous variables are summarized as medians with interquartile ranges, and categorical variables as counts and percentages. Group comparisons were performed using the Mann–Whitney U test for continuous variables and the χ^2^ test or Fisher’s exact test for categorical variables, as appropriate. Because KPS is an ordinal scale, within-group changes were evaluated using the Wilcoxon signed-rank test.

PFS and OS were estimated using the Kaplan–Meier method, and intergroup differences were assessed with the log-rank test. Median survival times and corresponding 95% confidence intervals (CIs) were reported.

To mitigate baseline imbalances between the NeoBev and Control groups, PSM was performed using a logistic regression model incorporating preoperative KPS and EOR. Nearest-neighbor matching (1:1) with a caliper width of 0.2 was applied, and covariate balance was evaluated using standardized mean differences before and after matching. A caliper width of 0.2 was selected in accordance with standard recommendations to reduce residual bias.

Cutoff values for MRI-based volumetric changes (T1Gd and FLAIR) were determined using ROC curve analysis, with optimal thresholds identified by the closest-to-(0,1) method (upper-left proximity).

In the matched cohort, effect sizes for survival outcomes were further quantified using Cox proportional-hazards models. Hazard ratios (HRs) with 95%CIs were estimated for the NeoBev group versus the Control group. Because matching produces paired observations, robust standard errors were used. All *p*-values were two-sided, and statistical significance was accepted for values of *p* < 0.05.

All analyses were conducted using R software (version 4.4.1; R Foundation for Statistical Computing, Vienna, Austria) and Stata 14 (StataCorp, College Station, TX, USA).

## 3. Results

### 3.1. Baseline Comparison Before and After PSM

Before matching, baseline characteristics were similar between the NeoBev (n = 33) and Control groups (n = 136), except for the extent of resection (EOR), which was significantly higher in the NeoBev group (*p* < 0.01). No significant differences were observed in age, sex, or preoperative KPS (Table 1). After 1:1 PSM, 33 patients were selected per group, and all prespecified covariates were well balanced (standardized mean differences < 0.10 and *p* > 0.10 for all; Table 2).

### 3.2. Functional Improvement After NeoBev Administration

**Figure 1:**
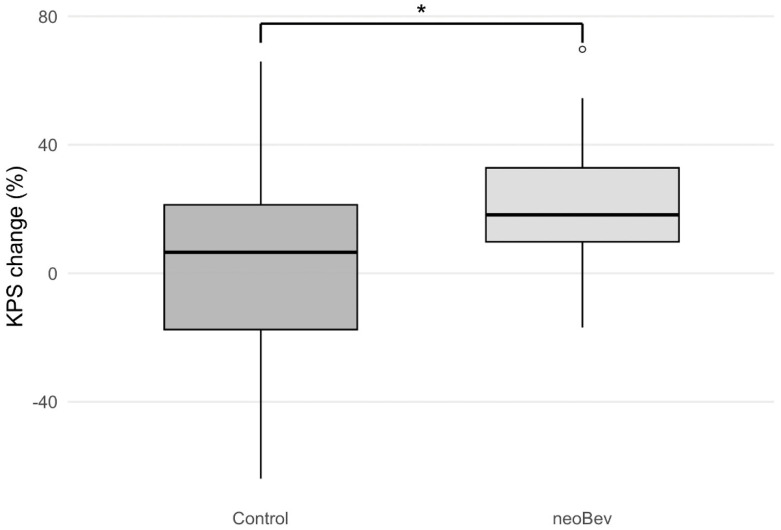
Postoperative improvement of KPS in the neoBev and control groups. Boxplot showing postoperative changes in Karnofsky Performance Status (KPS) in patients receiving preoperative bevacizumab (neoBev) compared with the control group. Within-group changes in KPS were evaluated using the Wilcoxon signed-rank test, and between-group differences in postoperative KPS change were assessed using the Mann–Whitney U test. Boxes represent the interquartile range (IQR), horizontal lines indicate the median, and whiskers denote 1.5 × IQR. Outliers are plotted as individual points. * = *p* < 0.05.

The functional impact of neoBev was assessed by comparing ΔKPS before and after surgery between the two groups. The NeoBev group displayed significantly greater improvement in postoperative KPS (+19.2%) than the Control group (+4.1%; *p* = 0.02) (Figure 1). This supports a potential role for neoBev in improving preoperative condition and perioperative functional status, although survival outcomes did not differ significantly between groups. No unexpected perioperative complications related to preoperative neoBev were observed. All patients in the neoBev group received a single preoperative dose as scheduled, with no protocol deviations. After adjustment for EOR, the association was attenuated but directionally consistent. Detailed results of percentage changes in KPS after surgery in the propensity score–matched cohort are provided (Supplementary Table S1).

### 3.3. Survival Analysis Before and After PSM

**Figure 2:**
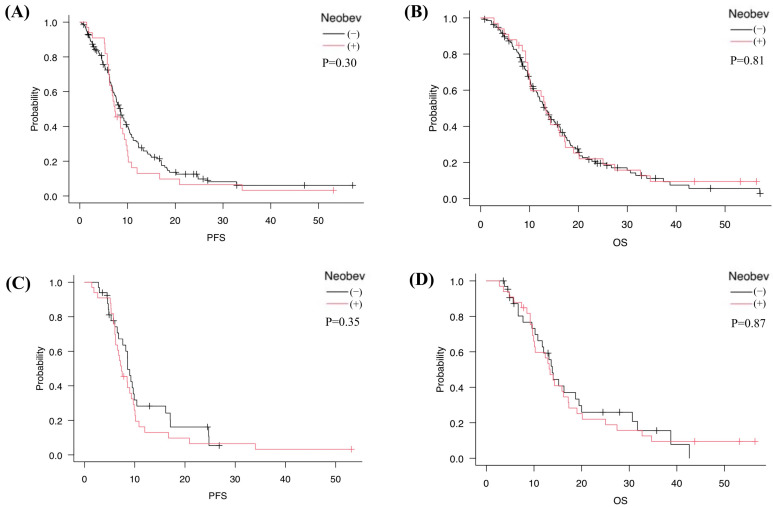
Kaplan–Meier analyses before and after PSM. Kaplan–Meier curves for PFS and OS comparing patients treated with neoadjuvant bevacizumab (neoBev) and those receiving standard therapy, shown before (**A**,**B**) and after (**C**,**D**) propensity score matching. Survival differences between groups were assessed using the log-rank test. Median survival times and corresponding *p*-values are shown for reference. Black line = neoBev (−); red line = neoBev (+). “(+)” indicates neoBev administration. “(−)” indicates no neoBev.

Before PSM, mPFS was 7.4 months in the NeoBev group and 8.4 months in the Control group (*p* = 0.30), while mOS was 13.4 months in the NeoBev group and 13.1 months in the Control group (*p* = 0.81). No significant differences were observed between the two cohorts (Figure 2A,B).

After 1:1 PSM, 33 patients were included in each group. Median mPFS was 7.4 months in the NeoBev group and 8.6 months in the matched Control group (*p* = 0.35). In comparison, mOS was 13.4 months in the NeoBev group and 13.8 months in the matched Control group (*p* = 0.87). Once again, no significant differences in survival were observed (Figure 2C,D). In the matched cohort, Cox proportional hazards analysis showed no significant difference in OS between the NeoBev and Control groups (HR 0.95, 95%CI 0.63–1.44; *p* = 0.81). Similarly, PFS did not differ significantly between groups (HR 1.24, 95%CI 0.83–1.85; *p* = 0.30), consistent with Kaplan–Meier analysis.

### 3.4. Exploratory Imaging Analyses (Refer to Supplementary Materials)

Exploratory analyses of early neuroradiographic changes after neoadjuvant bevacizumab were performed to generate hypotheses regarding imaging correlates of clinical outcomes. Therapeutic benchmarks from prior studies of standard concurrent radiotherapy and temozolomide were used as reference indicators in receiver operating characteristic (ROC) analyses. Post hoc ROC analyses identified candidate thresholds for volumetric reduction on contrast-enhanced T1-weighted MRI (T1Gd) and for FLAIR hyperintensity, which were subsequently applied for exploratory subgroup stratification (Supplementary Figure S1).

### 3.5. Association Between Imaging Changes and Survival

Exploratory survival analyses stratified by neuroradiographic volumetric changes are presented (Supplementary Figure S2 and Table 3 and Table 4). Stratification by FLAIR hyperintensity reduction did not show a significant association with progression-free survival (PFS) or overall survival (OS). In contrast, exploratory stratification by volumetric reduction on T1Gd MRI suggested associations with both PFS and OS. Patients demonstrating greater T1Gd volume reduction had more prolonged survival than those with smaller reductions. These neuroradiographic subgroup analyses were exploratory and should not be interpreted as evidence of a validated imaging biomarker.

### 3.6. Impact of EOR on Survival

Exploratory analyses stratified by extent of resection (EOR) were performed to descriptively assess how surgical extent may interact with perioperative treatment strategies. EOR-stratified Kaplan–Meier analyses for PFS and OS are shown (Supplementary Figure S3). Across EOR strata, no consistent survival advantage associated with neoadjuvant bevacizumab was observed. These subgroup analyses were not powered for definitive comparisons and are presented for descriptive purposes only.

## 4. Discussion

As previously described [1], neoadjuvant bevacizumab (neoBev) may reduce both tumor burden and perifocal edema before surgery, potentially facilitating safer resection and mitigating surgical morbidity, particularly in tumors located within or adjacent to eloquent brain regions. The safety profile of preoperative neoBev has been reported in detail in our prior prospective study [1], and no additional safety concerns were identified in the present secondary analysis.

Most prior clinical investigations of neoBev have focused on patients with unresectable glioblastoma, and, to date,, no studies have directly compared neoBev with the current standard-of-care regimen of maximal safe resection followed by concurrent radiotherapy and temozolomide. Because earlier cohorts differed substantially with respect to clinical background, tumor resectability, and surgical intent, direct comparisons with standard therapy are inherently challenging. Moreover, in patients with surgically accessible glioblastoma in whom gross total resection can be safely achieved, the incremental benefit of neoBev is expected to be limited, thereby making it particularly difficult to demonstrate a survival advantage in newly diagnosed disease.

Importantly, these earlier studies were primarily feasibility-oriented, single-arm investigations and therefore subject to selection biases related to tumor eloquence, patient age, and preoperative performance status. Building on these preliminary findings, the present study adopted a retrospective comparative design with propensity score matching (PSM) to more rigorously assess whether neoBev provides additional clinical benefit beyond standard chemoradiotherapy.

Given existing evidence that neoBev may transiently modulate the tumor microenvironment by alleviating hypoxia and normalizing tumor vasculature [9,10,11,12,16], we hypothesized that these changes could enhance the effectiveness of subsequent postoperative therapies, including radiotherapy and temozolomide. Accordingly, we evaluated the clinical impact of neoBev by comparing progression-free and overall survival between patients treated with neoBev and those receiving standard therapy, while accounting for relevant clinical factors using PSM.

### 4.1. Relationship to Prior Randomized Evidence and Clinical Positioning

Large randomized trials evaluating bevacizumab in newly diagnosed glioblastoma have consistently demonstrated prolongation of progression-free survival without a corresponding improvement in overall survival when administered in combination with standard chemoradiotherapy. This pattern is exemplified by the RTOG 0825 phase III trial, which showed that although bevacizumab extended PFS, it failed to confer an overall survival benefit compared with standard chemoradiation alone [17].

The results of the present study are broadly concordant with this established evidence. In the propensity score–matched cohort, preoperative neoadjuvant bevacizumab did not improve progression-free survival or overall survival compared with standard therapy, indicating that neoBev does not exert a generalized survival-prolonging effect in resectable glioblastoma. The observed hazard ratios for OS (HR 0.95, 95% CI 0.63–1.44) and PFS (HR 1.24, 95% CI 0.83–1.85) further support the absence of a clinically meaningful survival difference between treatment groups.

Exploratory imaging analyses suggested that patients exhibiting greater volumetric reduction on T1-weighted gadolinium-enhanced MRI tended to experience longer PFS and OS. However, these findings were derived from post hoc subgroup analyses and should be interpreted with caution. Rather than supporting routine use of neoBev, these observations suggest that any potential benefit of preoperative bevacizumab may be limited to carefully selected patients in whom perioperative factors—such as tumor eloquence, substantial edema, or hypervascularity—pose challenges to achieving safe and extensive resection.

Taken together, our findings reinforce the current clinical position that bevacizumab should not be considered a universal preoperative strategy for newly diagnosed glioblastoma, but may have a contextual role as a perioperative modifier in selected cases, without altering the overall disease trajectory.

### 4.2. Surgical Implications and EOR

Preoperative neoadjuvant bevacizumab (neoBev) has been hypothesized to facilitate more extensive surgical strategies, including supratotal resection or FLAIRectomy, by reducing tumor-associated edema and vascular permeability, potentially improving local tumor control in selected patients [18,19]. At the same time, concerns have been raised regarding treatment-related changes in tumor invasion patterns or distant dissemination following bevacizumab exposure. Prior studies have demonstrated that bevacizumab can effectively reduce peritumoral edema and vascular permeability in newly diagnosed glioblastoma, which may translate into improved surgical operability in carefully selected cases [20].

Direct histopathological confirmation of microscopic infiltration patterns after neoBev remains challenging, particularly in the context of supratotal resection or FLAIR-guided surgery for tumors located near eloquent brain regions. In this setting, analyses stratified by extent of resection (EOR) may offer descriptive insight into how perioperative treatment strategies interact with surgical feasibility. In patients for whom gross total resection was achievable, standard therapy yielded progression-free survival outcomes comparable to or numerically superior to those observed with neoBev, suggesting that preoperative bevacizumab is unlikely to confer additional benefit once near-complete resection can be safely achieved.

Conversely, in cases in which tumor location, mass effect, or vascularity preclude gross total resection—particularly for lesions adjacent to eloquent areas—neoBev may influence perioperative conditions by improving preoperative neurological status or surgical operability. In such contexts, subtotal or partial resection may be intentionally selected to minimize postoperative neurological deficits, even at the expense of maximal cytoreduction. Exploratory EOR-stratified analyses indicated a tendency toward longer overall survival in the neoBev group among patients undergoing subtotal or partial resection; however, this observation should be interpreted cautiously and may reflect preservation of postoperative functional status and treatment tolerability rather than a direct antitumor effect.

Importantly, these hypotheses require prospective validation using objective surgical endpoints, such as operative time, intraoperative blood loss, brain relaxation, and resection margins. The EOR-stratified findings in the present study were exploratory and did not reach statistical significance; therefore, they should not be interpreted as evidence of benefit or harm attributable to neoadjuvant bevacizumab.

### 4.3. Safety, Timing, and Perioperative Considerations

In the present protocol, surgical resection was scheduled 21–30 days after bevacizumab administration, balancing the potential risks of impaired wound healing and perioperative bleeding against the anticipated anti-edema and vascular-modulating effects of VEGF inhibition. Although the current study was not powered to evaluate perioperative safety outcomes formally, this timing strategy was selected to mitigate known risks while preserving the intended perioperative benefits of neoBev.

Our experience underscores the importance of an adequate washout interval, together with meticulous perioperative management, including careful hemostasis and wound care, when bevacizumab is used before surgery. Nevertheless, the absence of standardized safety endpoints in this analysis precludes definitive conclusions regarding perioperative risk.

Concerns regarding delayed wound healing and other surgical complications following preoperative VEGF inhibition have been reported in prior clinical series, highlighting the need for cautious surgical timing [21]. In addition, meta-analyses have demonstrated an increased risk of wound-healing complications and thromboembolic events associated with bevacizumab, further reinforcing the importance of an appropriate washout period before surgical intervention [22]. Future studies incorporating predefined safety endpoints will be essential for more accurately characterizing perioperative risks in this setting.

The 21–30-day interval between bevacizumab administration and surgery was chosen to minimize wound-healing complications while maintaining potential perioperative benefits. Given the approximately 20-day half-life of bevacizumab and the reported biphasic peaks of VEGF-mediated angiogenic activity during postoperative wound healing, this interval may represent a pragmatic compromise between therapeutic efficacy and surgical safety.

Although the optimal timing of surgery after bevacizumab remains to be established [1], our approach aligns with prior reports suggesting that a 3–4-week interval may reduce perioperative risks while preserving functional improvement. Prospective studies are needed to define the safest and most effective surgical window.

### 4.4. Imaging Biomarker Rigor and Cutoffs

A methodological strength of the present study lies in the use of predefined imaging time points and semi-automated volumetric assessments. To mitigate bias in a relatively small cohort, stepwise ROC curves were used to explore candidate thresholds for volumetric change, applying the closest-to-(0,1) criterion. In this exploratory framework, a greater reduction in contrast-enhanced tumor volume on T1-weighted gadolinium-enhanced MRI (T1Gd) was associated with improved survival. In contrast, a decrease in FLAIR hyperintensity demonstrated limited discriminative value.

Interestingly, this dissociation between contrast-enhancing and FLAIR-based responses warrants careful interpretation. Reduction in contrast-enhancing tumor volume has long been associated with prognosis in glioblastoma under established response criteria, such as the Macdonald and RANO frameworks, and therefore may reflect general tumor biology rather than a neoadjuvant bevacizumab-specific effect. In contrast, changes in FLAIR hyperintensity are more susceptible to modulation by anti-edema effects and vascular permeability, particularly under VEGF inhibition. The lack of a favorable survival association with marked FLAIR reduction in the present cohort suggests that dramatic FLAIR shrinkage after bevacizumab may not necessarily translate into oncologic benefit but rather reflects a complex composite of anti-edema and microenvironmental effects. This observation underscores the challenge of interpreting so-called “pseudo-response” phenomena in the era of anti-angiogenic therapy.

This differential pattern is consistent with the Response Assessment in Neuro-Oncology (RANO) criteria, which recognize “pseudo-response” as a rapid decrease in contrast enhancement without a corresponding reduction in infiltrative tumor burden [23]. Such imaging changes are thought to reflect early vascular normalization—characterized by reduced vascular permeability and edema—rather than true cytoreduction, a concept initially proposed by Jain [24]. Subsequent work by Sørensen et al. demonstrated substantial inter-patient variability in the magnitude and duration of this normalization process, as quantified by the vascular normalization index [25]. Together, these observations provide a plausible explanation for why changes in FLAIR signal predominantly reflect edema dynamics. In contrast, reductions in T1Gd-enhancing volume may better capture transient biological effects of bevacizumab exposure in selected patients.

Notably, the volumetric thresholds explored in this study were identified post hoc and should be regarded as hypothesis-generating rather than definitive imaging biomarkers. External validation in independent cohorts will be required, ideally incorporating advanced MRI techniques such as perfusion- and diffusion-weighted imaging, as well as radiomic approaches, to improve robustness and generalizability.

Emerging molecular and spatial profiling data suggest that variability in VEGF expression and its modulation following anti-VEGF therapy may contribute to heterogeneity in imaging responses and clinical outcomes. Within the conceptual framework of vascular normalization, anti-VEGF therapy is thought to transiently restore a more organized and functional microvasculature, thereby improving oxygenation and drug delivery while reducing edema [24,26,27]. However, increasing the intensity or duration of VEGF-targeted therapy has not been shown to produce durable survival benefits compared with cytotoxic modalities such as radiotherapy and temozolomide. Accordingly, changes in the tumor microenvironment during bevacizumab therapy may influence short-term disease control or functional status. In contrast, the mechanisms underlying rapid disease progression during or after VEGF inhibition remain incompletely understood.

Consistent with this interpretation, preoperative bevacizumab has been investigated across multiple solid tumor types, including rectal [28], hepatic [29], and renal cancers [30]. These studies have similarly reported transient tumor shrinkage and improved local resectability without sustained survival benefit. Collectively, these findings support the view that VEGF blockade primarily functions as a perioperative modifier—facilitating edema control and surgical feasibility—rather than as a strategy for prolonging overall survival, consistent with the observations of the present study.

### 4.5. Methodological Strengths

The present multi-institutional analysis incorporated several methodological features to enhance internal validity, including consistent MRI acquisition windows, semi-automated volumetric assessments, and propensity score matching to minimize baseline imbalances between treatment groups. In addition, exploratory responder analyses were conducted using ROC-derived candidate thresholds, enabling structured hypothesis generation while maintaining transparency about post hoc analyses. The concordance observed between progression-free survival and overall survival trends among patients with greater T1Gd volumetric reduction supports the internal consistency of the imaging findings, while acknowledging their exploratory nature.

From a clinical perspective, real-world neurosurgical practice often involves patients with substantial peritumoral edema or hypervascular tumors, in whom immediate surgical intervention may pose an elevated risk of neurological morbidity. In such settings, preoperative neoBev may influence perioperative conditions by improving neurological status or reducing surgical complexity, thereby facilitating safer operative strategies in selected cases.

Rather than serving as a therapy that maximizes outcomes in patients already eligible for gross total resection, neoBev may be more appropriately conceptualized as an intervention that mitigates extreme perioperative risk in patients with limited resectability. This interpretation emphasizes its potential contextual role in perioperative management without implying a generalized benefit across all surgically accessible glioblastoma cases.

### 4.6. Limitations

Several limitations of this study should be acknowledged. First, the retrospective design and relatively modest sample size limit statistical power and increase the potential for residual confounding, despite the use of propensity score matching to balance key baseline characteristics. Accordingly, causal inferences regarding treatment effects cannot be drawn.

Second, molecular profiling data, including methylguanine methyltransferase (MGMT) promoter methylation, telomerase reverse transcriptase (TERT) status, and epidermal growth factor receptor (EGFR) alterations, were incomplete in a subset of patients. Given the known prognostic and predictive relevance of these molecular factors, their absence may have influenced survival outcomes and treatment responsiveness, particularly to temozolomide-based therapy.

Third, heterogeneity in treatment exposure, including the use of a single preoperative dose of bevacizumab and institution-specific perioperative practices, may have contributed to variability in clinical outcomes. In addition, the volumetric cutoff thresholds used for exploratory imaging analyses were identified in a data-driven, post hoc manner and therefore require external validation in independent cohorts before any clinical application.

Because neoadjuvant bevacizumab was preferentially administered to patients with substantial edema, mass effect, or neurological deficits that increased surgical risk, an inherent selection bias toward clinically more challenging cases cannot be excluded. This limitation should be considered when interpreting functional and survival outcomes.

Taken together, these limitations underscore the need for prospective studies with standardized molecular characterization, predefined imaging endpoints, and systematic assessment of perioperative outcomes to more definitively define the role of neoadjuvant bevacizumab in newly diagnosed glioblastoma.

### 4.7. Future Directions

Future research should focus on identifying robust, reproducible prognostic or predictive biomarkers rather than relying on transient radiographic responses observed during short-term therapy. In this context, prospective biomarker-informed studies will be essential to validate exploratory imaging thresholds, such as the degree of T1-weighted gadolinium-enhanced (T1Gd) volume reduction, and to determine their clinical relevance.

Well-designed prospective trials could evaluate selection algorithms that integrate preoperative clinical factors—including tumor eloquence, edema burden, and vascularity—with molecular characteristics and quantitative imaging parameters. Randomized or adaptive study designs stratified by planned extent of resection and eloquent area involvement may help clarify whether neoadjuvant bevacizumab facilitates safer surgical strategies or improves perioperative functional outcomes without increasing surgical morbidity, including wound-healing complications.

In parallel, translational investigations using surgical specimens obtained after preoperative bevacizumab exposure may provide further insight into treatment-induced biological changes within the tumor microenvironment. Advanced neuroimaging modalities focusing on tumor oxygenation, such as (18)F-fluoromisonidazole positron emission tomography (FMISO-PET) [31], together with integrated molecular and multi-omics analyses, could contribute to a more comprehensive understanding of vascular normalization and its temporal dynamics. Such approaches inform the rational development of neoadjuvant strategies in glioblastoma.

## 5. Conclusions

Preoperative neoadjuvant bevacizumab did not improve progression-free survival or overall survival compared with standard chemoradiotherapy in this propensity score–matched analysis. Exploratory imaging analyses suggested that a greater reduction in contrast-enhanced tumor volume on T1-weighted gadolinium-enhanced MRI was associated with more favorable survival outcomes; however, these findings were hypothesis-generating and require external validation.

Taken together, the results indicate that neoadjuvant bevacizumab should not be considered a routine preoperative strategy for newly diagnosed glioblastoma. Its potential role may be limited to carefully selected patients in whom perioperative factors—such as extensive edema, lower preoperative performance status, or tumor involvement of eloquent regions—pose challenges to immediate surgical intervention, where optimization of perioperative conditions rather than survival prolongation is the primary clinical objective.

## Figures and Tables

**Table 1:** Baseline characteristics of the NeoBev and Control groups.

		Control	Neobev	*p* Value
Age	age < 60	38 (28%)	10 (30%)	0.83
	age ≥ 60	98 (72%)	23 (70%)	
	age < 75	93 (68%)	23 (70%)	1.00
	age ≥ 75	43 (32%)	10 (30%)	
EOR	PR	58 (43%)	5 (15%)	<0.01 *
	STR	34 (25%)	10 (30%)	
	GTR	44 (32%)	18 (55%)	
KPS	KPS ≤ 60	50 (37%)	13 (39%)	0.84
	KPS ≤ 70	86 (63%)	20 (61%)	
Location (left, right)	Left	65 (48%)	18 (55%)	0.78
	Right	62 (45%)	14 (42%)	
	Both	9 (7%)	1 (3%)	
Location(tent)	Supratentorial	132 (97%)	33 (100%)	1.00
	Infratentorial	4 (3%)	0	
Sex	Female	62 (46%)	10 (30%)	0.12
	Male	74 (54%)	23 (70%)	
IDH	Wildtype	108 (79%)	31 (94%)	1.00
	Mutation (+)	8 (6%)	2 (6%)	
	NOS	20 (15%)	0	
MGMT	Methylation (+)	26 (19%)	3 (9%)	0.71
	Unmethylation	27 (20%)	5 (15%)	
	NOS	83 (61%)	25 (76%)	

Bev = bevacizumab, EOR = extent of resection, GTR = gross-total resection, KPS = Karnofsky Performance Scale, NOS = not otherwise specified, PR = partial resection, STR = sub-total resection, * = *p* < 0.05. Values are shown as numbers (percentage within each group) unless otherwise indicated.

**Table 2:** Clinical characteristics of the NeoBev and Control groups (initial state and after propensity score matching).

		Initial State			Post Propensity Score Matching		
		Control	Neobev	*p* Value	Control	Neobev	*p* Value
Age	age < 60	38 (28%)	10 (30%)	0.83	9 (27%)	10 (30%)	1.00
	age ≥ 60	98 (72%)	23 (70%)		24 (73%)	23 (70%)	
	age < 75	93 (68%)	23 (70%)	1.00	21 (64%)	23 (70%)	0.79
	age ≥ 75	43 (32%)	10 (30%)		12 (36%)	10 (30%)	
EOR	PR	58 (43%)	5 (15%)	<0.01 *	5 (15%)	5 (15%)	1.00
	STR	34 (25%)	10 (30%)		10 (30%)	10 (30%)	
	GTR	44 (32%)	18 (55%)		18 (55%)	18 (55%)	
KPS	KPS ≤ 60	50 (37%)	13 (39%)	0.84	13 (39%)	13 (39%)	1.00
	KPS ≥ 70	86 (63%)	20 (61%)		20 (61%)	20 (61%)	
Sex	Female	62 (46%)	10 (30%)	0.12	11 (33%)	10 (30%)	1.00
	Male	74 (54%)	23 (70%)		22 (67%)	23 (70%)	

EOR = extent of resection, GTR = gross-total resection, KPS = Karnofsky Performance Scale, PR = partial resection, STR = sub-total resection, * = *p* < 0.05, Matching method: nearest-neighbor 1:1, caliper = 0.2.

**Table 3:** Relationship between volume reduction rate and median PFS in contrast-enhancing and edematous lesions based on the cutoff value.

Target	Decrease Rate	Number of Samples	Median PFS	95% CI (M)	*p* Value
Edematous lesion	Decrease rate < 55%	19	8.5	5.7–10.1	0.91
	Decrease rate ≥ 55%	14	6.5	5.3–9.7	
Contrast-enhanced lesion	Decrease rate < 37%	17	6.2	5.3–7.5	<0.01 *
	Decrease rate ≥ 37%	16	9.7	6.9–12.0	

CI = Confidence Interval, M = month, PFS = progression free survival, * = *p* < 0.05.

**Table 4:** Relationship between volume reduction rate and median OS in contrast-enhancing and edematous lesions based on cutoff values.

Target	Decrease Rate	Number of Samples	Median OS	95% CI (M)	*p* Value
Edematous lesion	Decrease rate < 55%	19	13.4	9.2–16.2	0.49
	Decrease rate ≥ 55%	14	14.1	9.2–25.0	
Contrast-enhanced lesion	Decrease rate < 37%	17	9.7	7.4–13.2	<0.01 *
	Decrease rate ≥ 37%	16	17.3	13.4–27.4	

CI = Confidence Interval, M = month, OS = overall survival, * = *p* < 0.05.

## Data Availability

The original contributions presented in this study are included in the article/Supplementary Materials. Further inquiries can be directed to the corresponding author.

## Supplementary Materials

The following supporting information can be downloaded at: https://www.mdpi.com/article/10.3390/cancers18030488/s1, Table S1: Percentage change in Karnofsky Performance Status (KPS)after surgery in the propensity score–matched cohort; Figure S1: Exploratory ROC analyses of volumetric response after neoadjuvant bevacizumab; Figure S2: Exploratory Kaplan–Meier analyses stratified by neuroradiographic volumetric changes after neoadjuvant bevacizumab; Figure S3: Exploratory analyses of survival according to extent of resection (EOR).

## Abbreviations

The following abbreviations are used in this manuscript:

VEGF	Vascular endothelial growth factor
Bev	Bevacizumab
GB	Glioblastoma
FLAIR	Fluid-attenuated inversion recovery
neoBev	Neoadjuvant bevacizumab
T1-Gd	T1-weighted imaging with gadolinium enhancement
mOS	Median overall survival
RT	Radiation therapy
TMZ	Temozolomide
PFS	Progression-free survival
MRI	Magnetic resonance imaging
EOR	Extent of resection
GTR	Gross total resection
