# Supplementary material for: Validation of Preoperative Neoadjuvant Bevacizumab Therapy for Newly Diagnosed Glioblastoma via Comparative Analyses with Propensity Score Matching
Source: Cancers (Basel). 2026 Feb 1;18(3):488. doi: 10.3390/cancers18030488 (PMC12897004; doi:10.3390/cancers18030488)
Supplement: Supplementary file 1 [file cancers-18-00488-s001.zip › cancers-4122189-Table S1.pdf]

Supplementary Table S1. Percentage change in Karnofsky Performance Status (KPS)after surgery in the propensity score–matched cohort

Group	n	KPS change (%) (mean ± SD)	p value
Control	33	2.9 ± 29.6	0.02
NeoBev	33	19.2 ± 23.3	

KPS change (%) was calculated as (postoperative KPS − preoperative KPS) / preoperative KPS × 100. Positive values indicate functional improvement, and negative values indicate functional deterioration. Comparisons were performed using Student's t-test (two-sided). *= $P<0.05$, SD=standard deviation,
