# Supplementary figures and images for: Validation of Preoperative Neoadjuvant Bevacizumab Therapy for Newly Diagnosed Glioblastoma via Comparative Analyses with Propensity Score Matching
Source: Cancers (Basel). 2026 Feb 1;18(3):488. doi: 10.3390/cancers18030488 (PMC12897004; doi:10.3390/cancers18030488)

Supplemental  
Figure S1

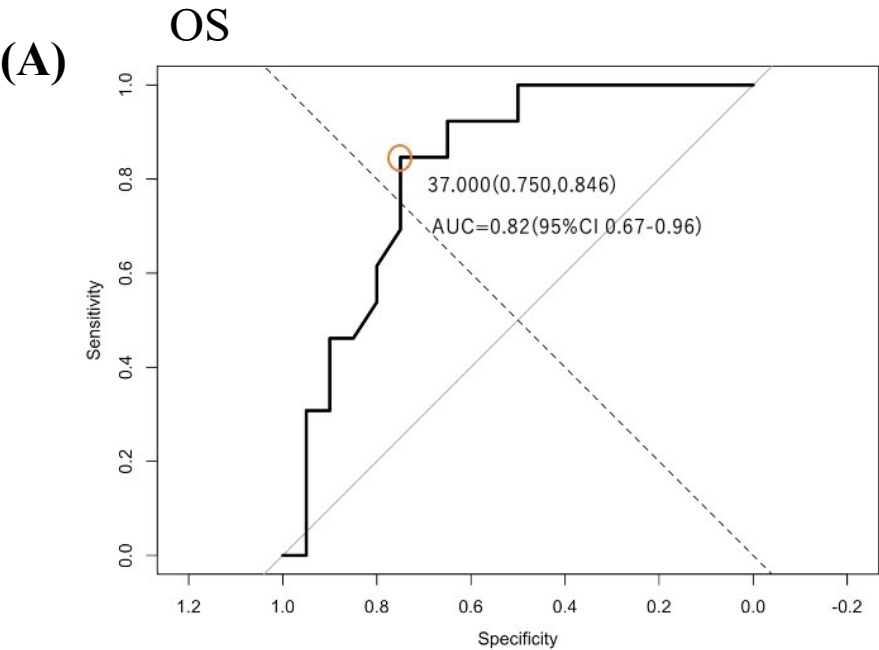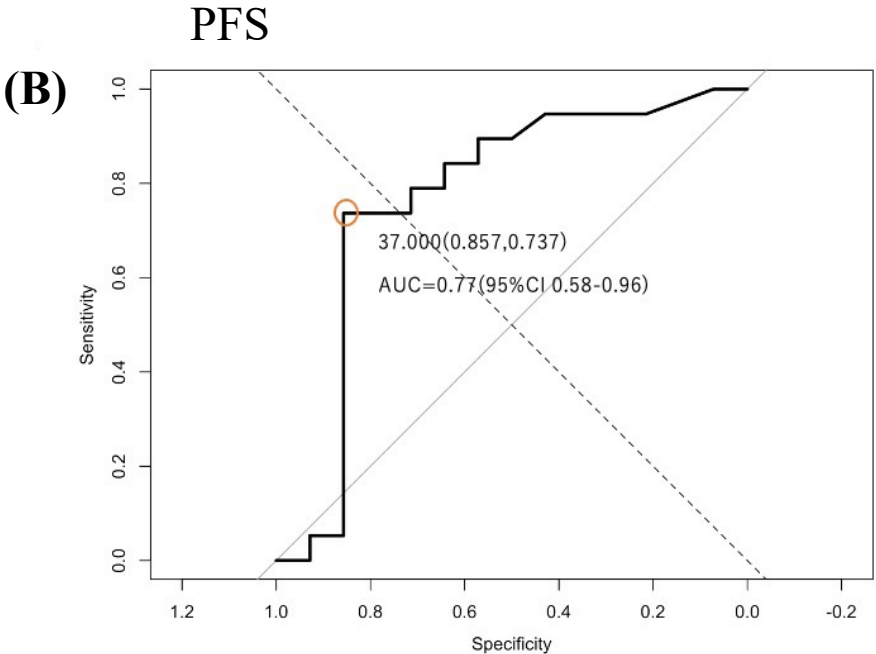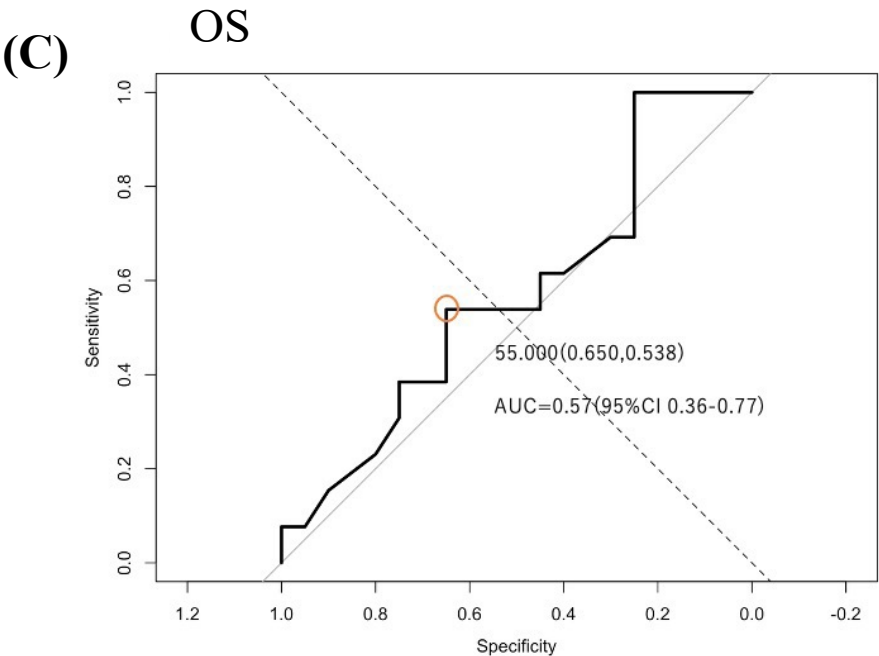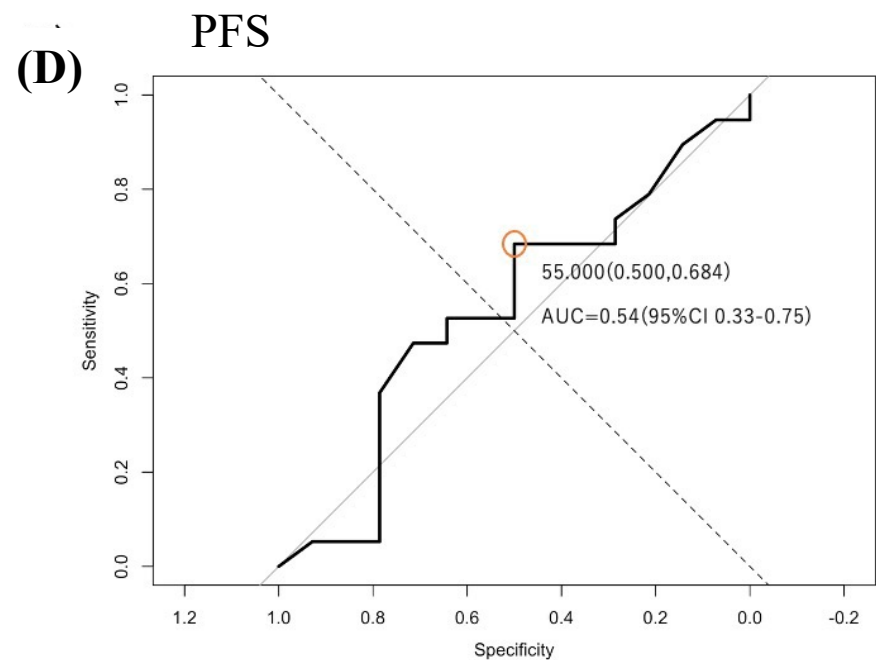

Supplemental  
Figure S2

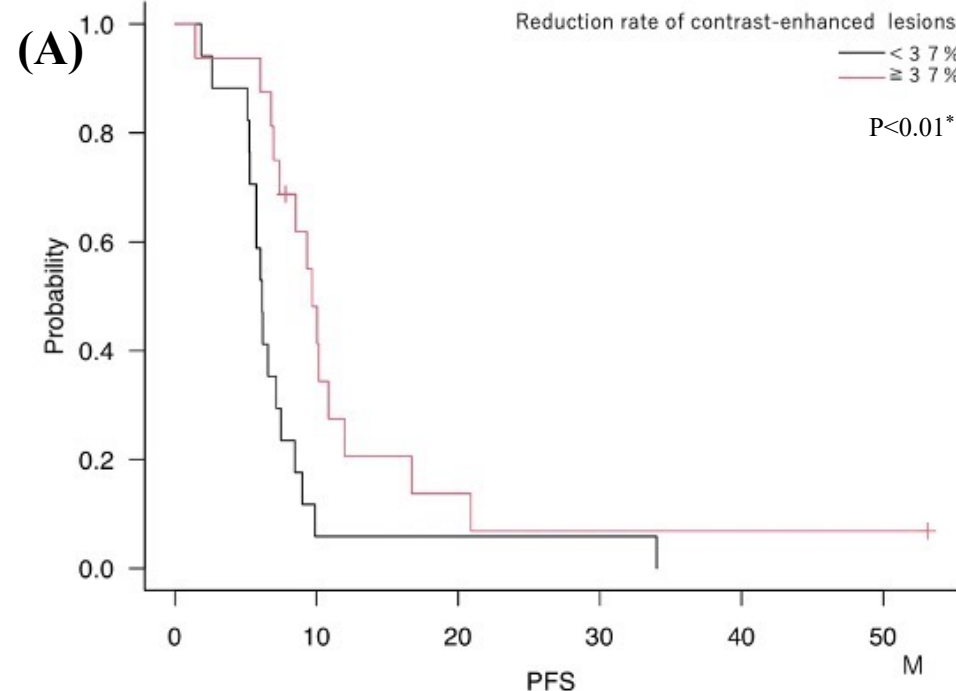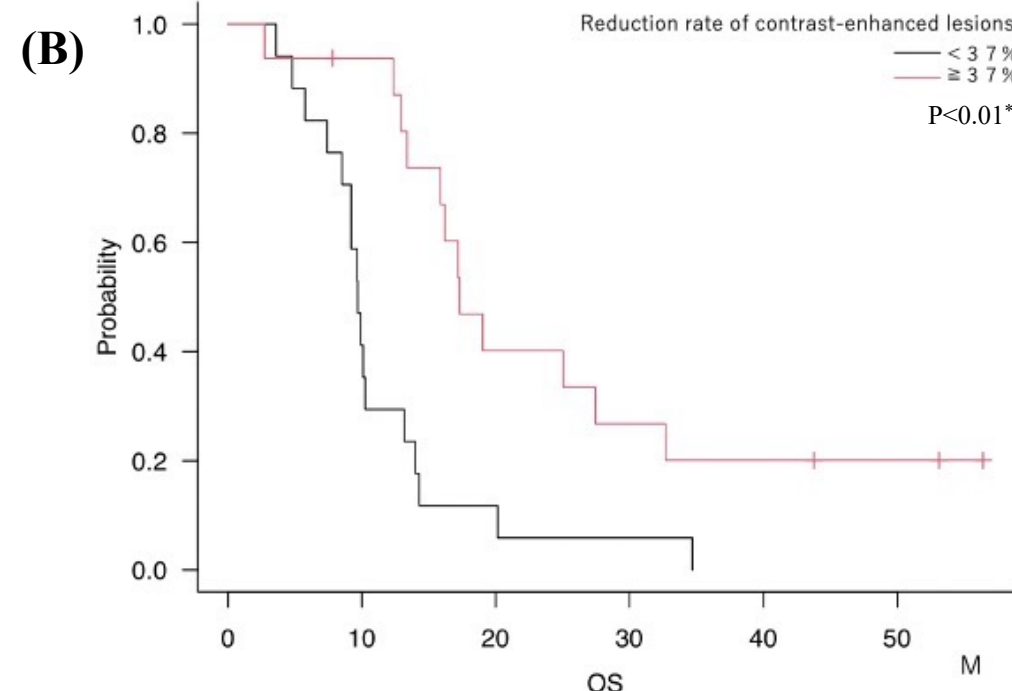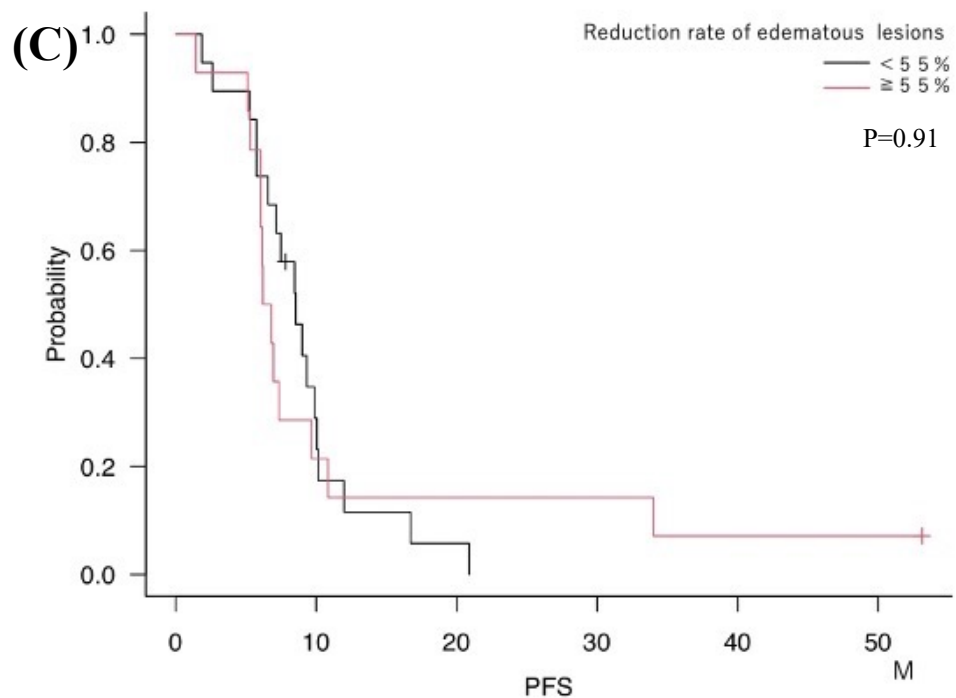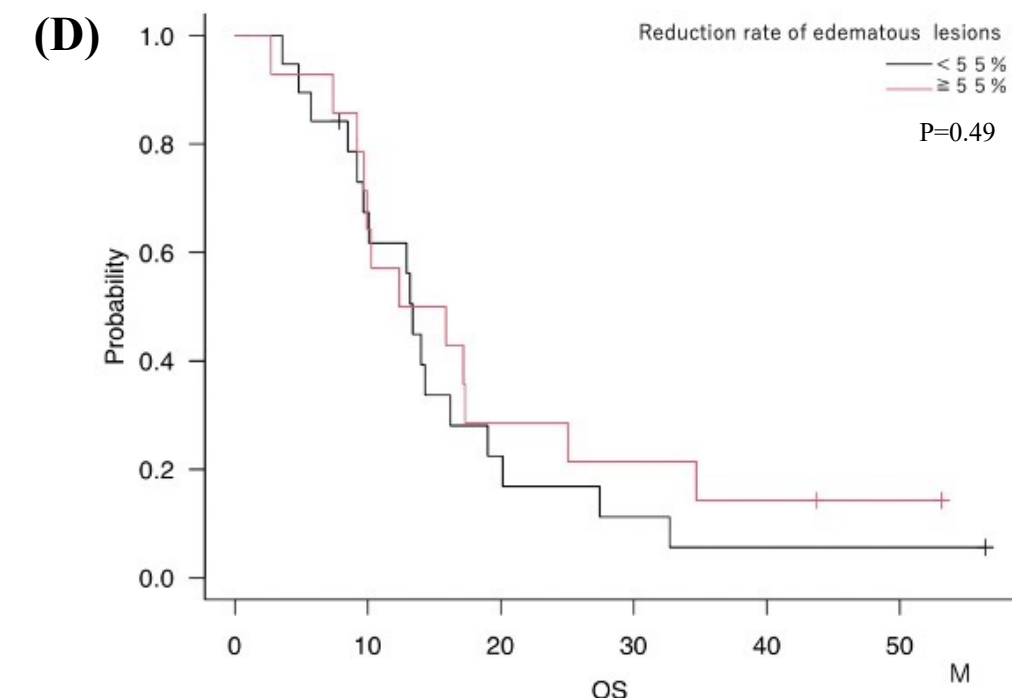

Supplemental  
Figure S3

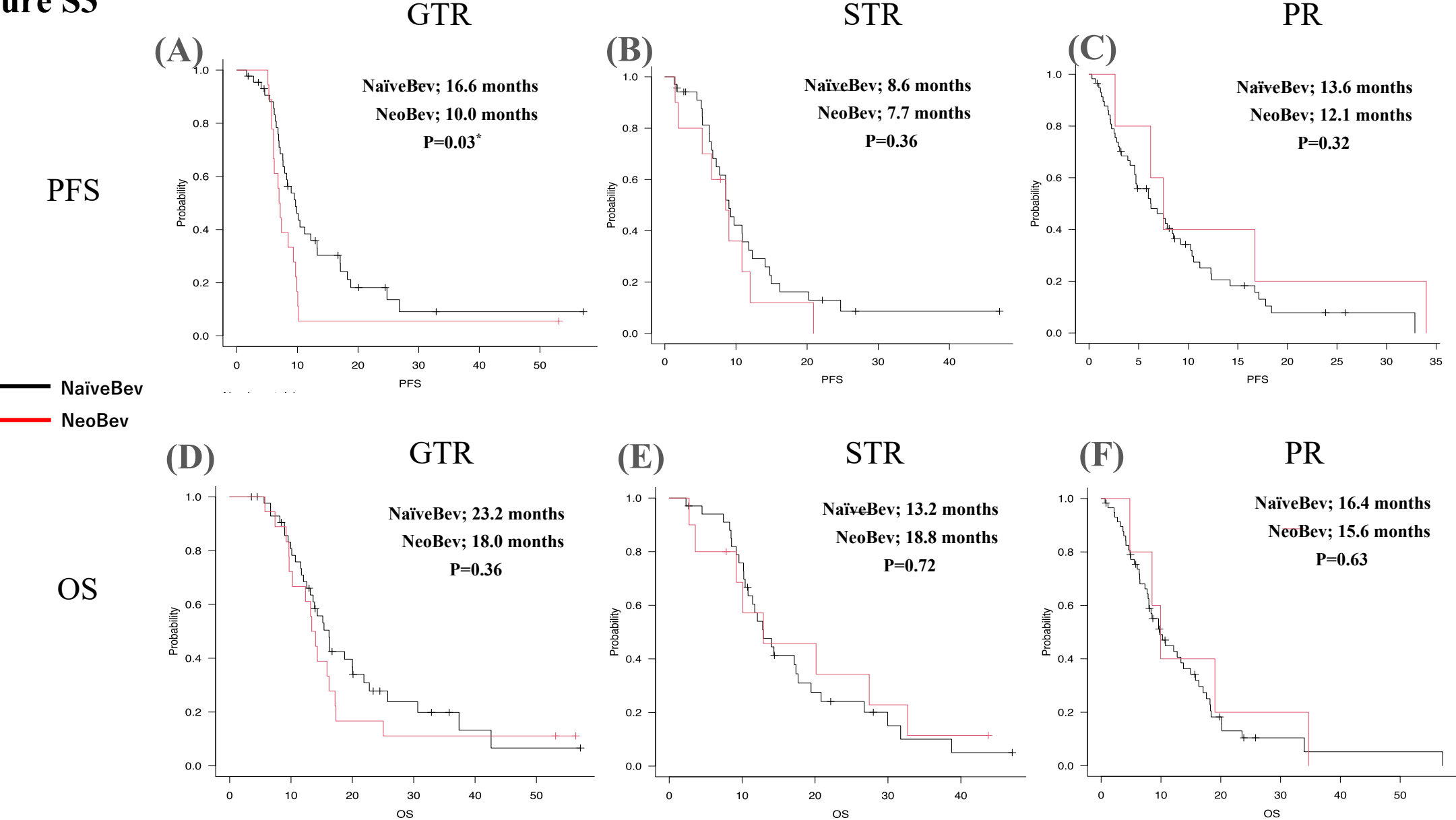

Supplement: Supplementary file 1 [file cancers-18-00488-s001.zip › cancers-4122189-Figures.pdf]
